# Acupotomy combined with massage for cervical spondylotic radiculopathy: A protocol for systematic review and meta-analysis

**DOI:** 10.1097/MD.0000000000021587

**Published:** 2020-08-07

**Authors:** Wenkang Dai, Xiongwei Wang, Rui Xie, Minghui Zhuang, Xiaojuan Chang, Zhefeng Jin, He Yin, Minshan Feng, Xu Wei, Jie Yu, Liguo Zhu

**Affiliations:** a Wangjing Hospital of China Academy of Chinese Medical Sciences; b Beijing University of Chinese Medicine, Chaoyang District, Beijing, China.

**Keywords:** acupotomy, cervical spondylotic radiculopathy, massage, meta-analysis, protocol

## Abstract

**Background:**

Cervical spondylotic radiculopathy (CSR) is a clinical syndrome of radial neck and shoulder pain. Both Massage and Acupotomy have been widely used in the treatment of CSR, in China and achieved satisfied efficacy. Therefore, the aim of this study is to systematically evaluate the clinical efficacy of acupotomy combined with massage in the treatment of CSR.

**Methods:**

The following electronic databases will be searched: PubMed, Web of Science, the Cochrane Central Register of Controlled Trials (CENTRAL), the Cochrane Library, Embase, SinoMed, Clinical Trials. gov, the China National Knowledge Infrastructure (CNKI), Wanfang database, and VIP database. Two review authors independently search databases from their respective inception dates to September 2019 to identify potentially eligible studies. Cochrane Handbook 5.1 risk of bias assessment tool will be used to evaluate the methodological quality of the included studies. The Review Manager 5.3 will be used for all statistical analysis of the final included study.

**Results:**

The results of this systematic review and meta-analysis will provide a synthesis of existing evidences for the treatment of acupotomy combined with massage on CSR, especially in improving visual analog scale and symptom score.

**Conclusion:**

This study will summarize the current evidence of acupotomy combined with massage for the treatment of CSR. This study can further guide the promotion and clinical decisions.

**Ethics and dissemination:**

Ethical approval and patient consent are not required because this study is a literature-based study. This systematic review and meta-analysis will be published in a peer-reviewed journal.

**PROSPERO registration number:**

CRD42020171825.

## 1 Introduction

Cervical spondylotic radiculopathy (CSR), which is pain, weakness or reduced reflexes that follow the path of nerves that come from the neck region.^[1]^ In recent years, it shows a younger and professional trend, which seriously affects the quality of life of patients.^[2,3]^

Management of CSR includes surgical and nonoperative treatment. Usually, patients should be treated conservatively for at least 6 weeks, surgical intervention should be considered for patients with no improvement after 6 to 12 weeks of nonoperative treatment, obvious muscle weakness, progressive neurological deficit or myelopathy. Nonoperative treatment is the first choice for most patients because of surgery-related adverse events and recurrence. Seventy five percent to 90% of patients achieving symptomatic improvement with nonoperative treatment.^[4,5]^ Currently, nonoperative treatments consist of anti-inflammatory pharmacotherapy, physical therapy, epidural steroid injections manipulation, cervical traction, exercise, acupotomy, massage, and various combinations of these.^[6,7]^

Acupotomy, also named needle knife, which resembles both Chinese acupuncture therapy and modern surgical principles, is a Traditional Chinese Medicine intervention widely used to treat neck pain and other diseases.^[8–10]^

Some hypotheses has been suggested that needle knife is to release the diseased tissue, relieve the stimulation or compression of nerves and blood vessels, and restore the ameliorate pain and numbness symptoms.^[11,12]^ Other studies have suggested that needle knife induces natural opioid-mediated pain suppression by stimulating local α-delta nerve fibers.^[13]^

Compared with the open surgery, acupotomy/needle knife has small wounds, no suture is needed, almost no tissue damage, and no obvious pain. Thus acupotomy reduces risk, time, and cost.^[14]^ acupotomy/needle knife has been widely used in clinical treatment of cervical spondylosis in China, and remarkable clinical effect has been obtained.

However, some scholars have found that some patients treated with acupotomy/needle knife alone can not achieve the desired results, so they need to be combined with other conventional treatments.^[15,16]^

The guidelines do not recommend acupotomy/needle knife combined with massage for the treatment of CSR.^[7]^

The aim of this study is to systematically evaluate the clinical efficacy of acupotomy/needle knife combined with massage in the treatment of CSR. Provide evidence of evidence-based medicine for clinical practice.

## 2 Methods

### 2.1 Study registration

This systematic review and meta-analysis protocol was registered in International Prospective Register of Systematic Reviews (PROSPERO) (registration no.CRD42020171825, which is available on https://www.crd.york.ac.uk/prospero/display_record.php?ID=CRD42020171825). Reviews and Meta-Analyses (PRISMA) approach and reported adhering to the guidelines.^[17]^

### 2.2 Inclusion criteria for study selection

#### 2.2.1 Type of studies

We will include randomized controlled trials; the exclusion criteria were shown as follows: review, case reports, cohort studies, expert experience, animal or cell experiments, and other non-RCTs; There are no restrictions on languages.

#### 2.2.2 Type of participants

Patients should have been diagnosed with CRS based on past or current guidelines for the diagnosis of CRS. We will include studies that not limited by age, sex, race, nationality.

#### 2.2.3 Type of interventions

This review is confined to RCTs comparing acupotomy combined with massage with a control group, which contained massage or tuina or chiropractic therapy; no restrictions are imposed on times of treatment, frequency of treatment, and length of treatment period; other types of acupuncture including fine needles, fire needling, electronic needling, ear auricular pressure treatment, acupoint pressure, and so forth are excluded.

#### 2.2.4 Types of outcome measurements

VAS, symptom score and total effective rate are used as outcome indicators. Changes in the visual analog score (VAS) and symptom score will assess patients before and after treatment. Participants are generally classified as “cured”, “markedly improved”, “improved” or “non-responder” after treatment. Total effective rate = X_1_+X_2_+X_3_ /X; X_1_, X_2_, X_3_ and X are the number of cured, markedly improved, improved patients and the total number of patients with sample size, respectively.

### 2.3 Search methods

#### 2.3.1 Data sources

The following electronic databases will be searched: PubMed, Web of Science, the Cochrane Central Register of Controlled Trials (CENTRAL), the Cochrane Library, Embase, SinoMed, Clinical Trials. gov, the China National Knowledge Infrastructure (CNKI), Wanfang database and VIP database. RCTs of acupotomy combined with massage vs massage for patients with CSR will be searched in the databases from their inception to September 2019 by 2 researchers independently.

#### 2.3.2 Search strategy

The Medical Subject Headings (MeSH), text words, and word variants for “nerve root cervical spondylotic”, “cervical spondylosis”, “cervical spondylotic radiculopathy”, “cervical spondylopathy”, “cervical syndrome”, “neck pain”, “mechanical neck disorders “, “acupotomy”, “needle knife”, “ scalpel needle”, “scalpel needling”, “miniscalpel needling”, “miniscalpel needle”, “stiletto needle”, “sword like needle”, “Xiaozhendao”, “acupotomology”, “massage”, “tuina”, “ Chiropractic therapy”,“clinical trials”, “randomized”, “randomised” are used and combined in the searches.

Different search strategies will be used for the different language databases.

There will be no limitation on language and publication type.

### 2.4 Selection of studies

We will use EndNoteX9 software to manage the retrieved literature.

The 2 researchers will independently search the literature, eliminate the repeated literature, read the title and abstract to screen the literature, exclude the literature that obviously does not meet the inclusion criteria, and further download the literature that may meet the criteria and re-screen the full text to determine whether it is included or not. Any disagreement will be resolved by consensus or consultation with a third independent researcher.

**Figure 1:**
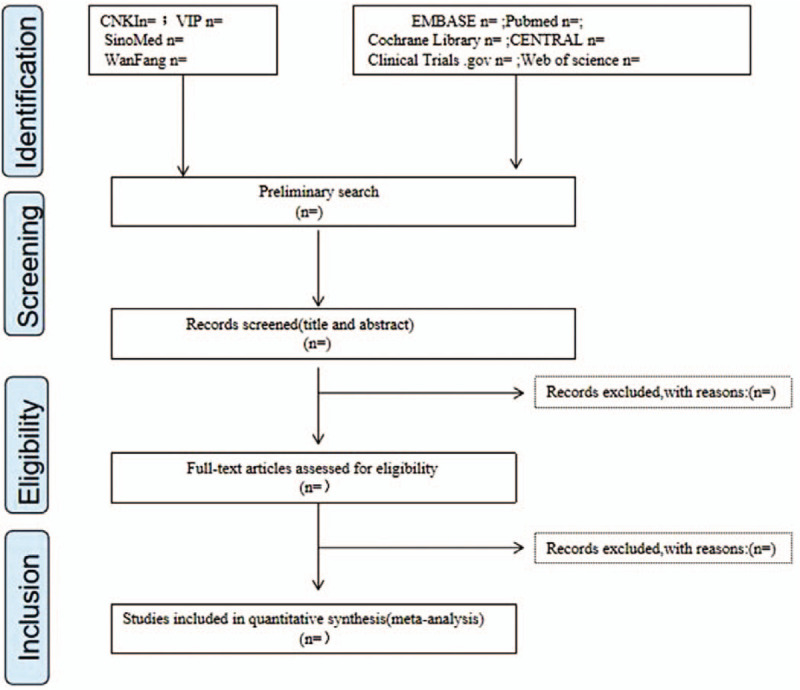
Flow diagram of study selection and screening process.

The selection process will be presented in a PRISMA flow diagram (Fig. 1).

### 2.5 Data extraction and management

Two researchers will independently complete the data extraction work included in the literature. We will make a table of the following information before data extraction: the publication time of the literature, the name of the first author, gender, age, sample size, intervention measures, outcomes, and follow-up time of the experimental group and the control group. The 2 researchers resolved their differences through consultation or with the third reviewer.

### 2.6 Assessment of risk of bias in included studies

Two of our independent reviewers will use RevMan 5.3 software (Copenhagen, The Nordic Cochrane Centre, The Cochrane Collaboration, 2014) provided by the Cochrane Collaboration^[18]^ to evaluate the quality and risk of bias in the ultimately included studies. Risk of bias assessment includes 7 important items:

1. random sequence generation;
2. allocation concealment;
3. blinding of participants and personnel
4. blinding of outcome evaluation;
5. incomplete report outcome;
6. selective outcome reporting;
7. other biases.

Make “Low risk”, “High risk”, and “unclear risk” judgments for each items. The evaluation results will be presented as a risk of bias graph and risk of bias summary. If inconsistent results are obtained from the 2 reviewers, a third reviewer will intervene, and consensus will be reached by discussion.

### 2.7 Data analysis

RevMan 5.3 software (Copenhagen, The Nordic Cochrane Centre, The Cochrane Collaboration, 2014) will be used for meta-analysis. Dichotomous outcomes will use relative risk (RR) as the effect analysis statistics. Continuous outcomes will use mean difference (MD) as the effect analysis statistics. Both Dichotomous data and Measurement data will use the effect amount and its 95% confidence interval (CI). If the same outcome is measured by different ways, the standardised mean difference (SMD) with 95% CI will be used to express the size of the intervention effect. Statistical heterogeneity will be evaluated by *I*^2^ statistics and χ^2^ test. If *P* > .1 or *I*^2^ ≤ 50%, we will use a fixed effect model for meta-analysis; If *P* < .1 or *I*^2^ > 50%, we will analyze data using a random-effects model.

### 2.8 Sensitivity analysis

If there is significant heterogeneity, we will analyze the source of the heterogeneity.

### 2.9 Reporting bias analysis

If >10 qualified studies are included in our study, funnel plots will be used to assess the potential reporting biases. Harbord modified test and Egger test will be used to assess the publication bias.

## 3 Discussion

Cervical spondylotic radiculopathy (CSR) is common public health concerns in the world. Acupotomy, Massage and various combinations are widely used in the treatment of CSR in china.

Acupotomy, length ranges from 6 to 15 cm, diameter ranges from 0.4 mm to 1 mm, resembles both needle and knife in shape. Acupotomy is used for the treatment of CRS by directly piercing the touchable trigger point or pathologic sensitive point to strip the adhesion, remove the attached tissue and relieve the nerve pressure.^[19]^

Massage is used for the treatment of CRS by relieving the muscle spasm of neck, shoulder and back and restoring the biomechanical balance of cervical vertebra.^[20]^

Acupotomy and Massage, which contribute to pain and symptom relief, has gained popularity through promising results. In clinical practice, many patients with CRS will improve with acupotomy and massage care, although there are short of highquality literature to support the results. Moreover, the guidelines for acupotomy combined with massage therapy for CRS have not clear recommendations or weak recommendations due to lacking of high quality literature.^[7]^

Then, we hope the results of this systematic review and meta-analysis can help to propose the clinical recommendation for CRS and to provide more evidence about the application of acupotomy combined with massage therapy.

## Author contributions

**Conceptualization:** Wenkang Dai, Jie Yu.

**Data curation:** Xiongwei Wang, Rui Xie, Xu Wei.

**Investigation:** Minghui Zhuang, Xiaojuan Chang.

**Methodology:** Zhefeng Jin, He Yin, Xiongwei Wang.

**Resources:** Rui Xie, Minghui Zhuang.

**Software:** Xiaojuan Chang, Minshan Feng, Xu Wei.

**Supervision:** Jie Yu, Liguo Zhu.

**Writing – original draft:** Wenkang Dai.

**Writing – review & editing:** Wenkang Dai, Jie Yu, Liguo Zhu.
